# Patient registries in orthopedics and orthobiologic procedures: a narrative review

**DOI:** 10.1186/s12891-022-05416-4

**Published:** 2022-06-06

**Authors:** Cedric Lester Magaway, Gerard Malanga

**Affiliations:** 1 Rutgers Robert Wood Johnson Medical School, 125 Paterson St, New Brunswick, NJ 08901 USA; 2 New Jersey Regenerative Institute, 197 Ridgedale Ave #210, Cedar Knolls, NJ 07927 USA; 3 Clinical Professor Department of Physical Medicine & Rehabilitation, Rutgers New Jersey Medical School, 185 S Orange Ave, Newark, NJ 07109 USA

**Keywords:** Musculoskeletal Conditions, Orthobiologics, Outcomes Assessment/Measurement

## Abstract

There has been increasing evidence and growing popularity of orthobiologic treatments, such as platelet-rich plasma, bone marrow aspirate concentrate, and microfragmented adipose tissue. However, real-world data, including patient-reported pain and function outcomes, remains sparse for these procedures. Thus, collecting patient-reported outcome measures is important to evaluate the safety and efficacy of these treatments and hopefully improve patient care. Patient reported outcome measures can systematically be collected through patient registries. This narrative review serves to describe the data collection platforms and registries that obtain patient-reported outcome measures on orthobiologic procedures and provide a discussion on the benefits and limitations of registries. An internet search of the list of orthopedic registries available was conducted, and registries that collect patient-reported outcome measures for orthobiologic procedures were identified. Additional information regarding these various registries was collected by directly contacting these vendors. Publications from these registries, including case series, observational studies, and annual reports, were also reviewed. Providing this review will inform clinicians of a digital tool that can increase the efficiency of collecting outcome measures for orthobiologics and aid physicians in choosing a data collection platform.

## Background

Randomized controlled trials (RCTs) are the “gold standard” for evaluating the safety and efficacy of new therapeutic agents and medical interventions. RCTs achieve internal validity by reducing bias and confounding factors through randomization and strict patient inclusion and exclusion criteria. However, this often comes at the expense of external validity (generalizability) [1]. In addition, RCTs generally require extended periods of data collection and can be costly to perform. Real-world data is gathered outside of the conventional clinical trial setting and includes data obtained from patient charts, laboratory reports, patient registries, surveys, and mobile health devices [2]. This data can complement evidence obtained from RCTs by providing information about the long-term safety and effectiveness of medical interventions in large populations in a more naturalistic setting as well as allowing stakeholders and health insurance companies to assess the risk-benefit and economic value of medical interventions [1–3]. This allows for a more time-efficient and cost-effective method of data collection that is likely more reflective of the true clinical situations in which these procedures are performed. One method to systematically collect real-world data is through a registry database.

A registry is an organized system designed to collect uniform data to evaluate specific patient reported outcome measures (PROMs) for a population defined by a particular disease, condition, or exposure and which serves scientific, clinical, or policy purposes. PROMs are validated questionnaires that allow patients to report on their own health directly without interpretation from a physician [4]. Commonly employed PROMs include generic or general health instruments, which aim to provide a measure of general health for any health state [5]. An example is the European Qualify of Life (EurQol) 5 dimension health outcome survey (EQ-5D), which provides measures in the dimensions of mobility, self-care, usual activities, pain/discomfort, and anxiety/depression [4, 6]. The second main type of PROMs are specific instruments, which focus on a specific symptom, disease, organ, body region, or body function. These may also be designed to measure the effect of a specific intervention or treatment [5]. There are numerous body-specific PROMs that have been validated and widely used for orthopedic conditions, such as the Knee Injury and Osteoarthritis Outcome Score (KOOS) and Hip Outcome Score (HOS) [7–9].

The burden of musculoskeletal diseases is expected to increase in the near future. For example, it is estimated that the prevalence of self-reported, doctor-diagnosed arthritis is projected to increase from 47.8 million in 2005 to nearly 67 million by 2030, which is 25% of the adult population in the US. By 2030, 25 million or 9.3% of the US adult population is projected to report arthritis-attributable activity limitations [10]. Furthermore, healthcare costs continue to rise in the US. As of 2018, the total health expenditure in the US was $3.6 trillion, which was 17.7% of the US gross domestic product [11]. With increasing prevalence of musculoskeletal diseases and growing healthcare costs, there has been a trend towards value-based health care and the need to systematically collect data in the form of patient registries. Orthobiologic procedures, such as platelet-rich plasma (PRP), bone marrow aspirate concentrate (BMAC), and microfragmented adipose tissue (MFAT), utilize biologically-derived substances to promote the healing of tissue for various orthopedic conditions [12, 13]. Various RCTs and case series have recently been published to help support their use, such as PRP for osteoarthritis of the knee and various tendinopathies [14–18]. There is less robust evidence for bone marrow and adipose tissue procedures. Although there is a lack of robust evidence for these procedures, they are becoming increasingly offered by physicians to treat various orthopedic conditions. PROMs collected through patient registries can help complement data from RCTs by evaluating the safety and efficacy of these orthobiologic treatments. Furthermore, data from PROMs could help demonstrate potential cost savings of orthobiologics as an alternative to current standards of care including surgery.

**Fig. 1:**
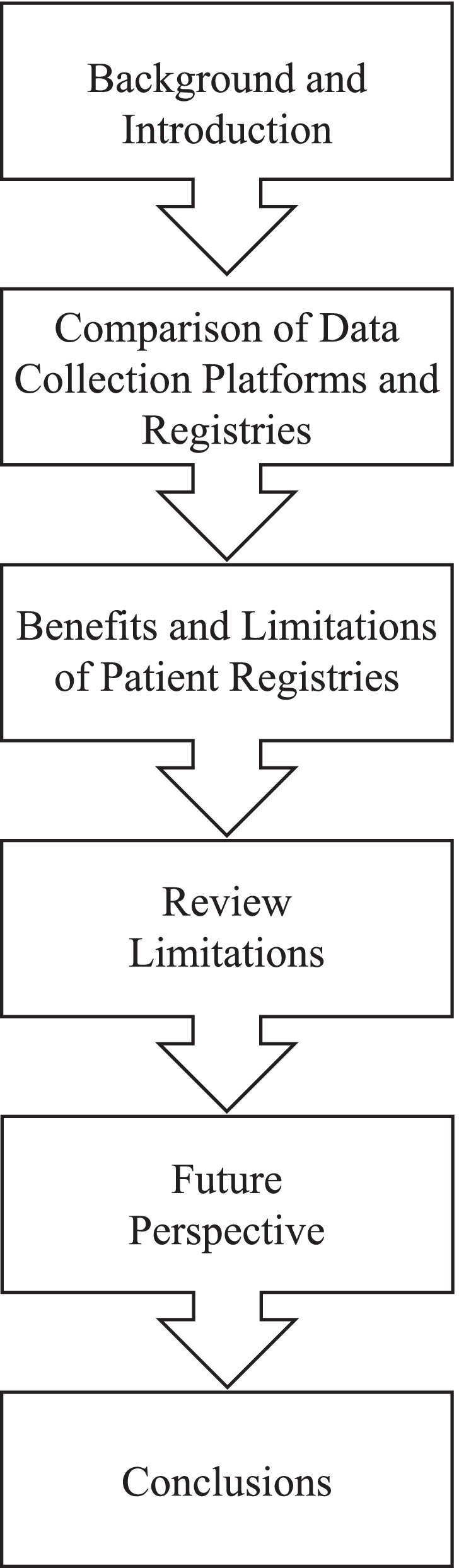
Summary of Review

This article serves to provide an overview and comparison of the available registries that collect data on orthobiologic procedures, such as PRP, BMAC, MFAT, allografts, and scaffolds. The characteristics of registries that will be discussed includes services provided by the registry, such as follow-up personnel or automatic reminders, platforms that patients can complete PROMs, HIPAA compliance, and costs. By providing this information, medical providers can obtain a better understanding of the registries and services available to collect PROMs that could potentially be used in their clinical practices. For this review, an internet search of the list of orthopedic registries available was conducted, and registries that collect PROMs for orthobiologic procedures were identified. Additional information regarding these various registries was collected by directly contacting these vendors. Publications from these registries, including case series, observational studies, and annual reports, were also reviewed. This review also provides a discussion on the limitations and benefits of registries as well as proposes methods to overcome challenges of using registries. A summary of this review can be found in Fig. 1 below.

## Main Text

### Registry systems

Orthopedic registry systems have mostly focused on joint replacements as well as other orthopedic surgical procedures. There are currently 31 members of the International Society of Arthroplasty Registries (ISAR), including Canada, Spain, Egypt, Germany, Switzerland, Sweden, India, Iran, Ireland, Italy, Japan, United Kingdom, Pakistan, Portugal, France, South Africa, Australia, Denmark, Netherlands, Finland, Lithuania, New Zealand, Norway, Romania, and Slovakia [19]. Several US national registries have also been developed, including the American Joint Replacement Registry (AJRR), Function and Outcomes Research for Comparative Effectiveness in Total Joint Replacement (FORCE-TJR), the Kaiser Permanente National Total Joint Replacement Registry (TJRR), the Veterans Affairs and American College of Surgeons National Surgical Quality Improvement Programs (NSQIPs), and the National Trauma Data Bank (NTDB) [20]. ArthritisPower is another patient registry that was funded through a Patient-Centered Outcomes Research Institute Award and jointly developed by the non-profit Global Healthy Living Foundation (GHLF), CreakyJoints arthritis patient community, and rheumatology researchers at the University of Alabama at Birmingham (UAB). It focuses on rheumatoid conditions, such as rheumatoid arthritis, psoriatic arthritis, and ankylosing spondylitis and has published in journals, such as *Arthritis Research & Therapy*. However, it tracks response to medications, such as methotrexate, rather than orthobiologic procedures [21, 22]. The following data collection platforms and registries will be focused on those that collect outcomes for orthobiologic procedures. A summary of these platforms and registries is provided in Table 1 and Table 2.

**Table 1:** Summary of Orthobiologics and PROMs

Registry/Data Collection Platform	Orthobiologic Procedures	Patient Reported Outcome Measures Used		HIPAA Compliant
Oberd – Regenerative Orthobiologics Registry (ROR)	A2M Amnion Matrix Allograft Fibrin Matrix Cord Allograft Plasma Concentrate Platelet Lysate PPP PRP Stem Cells	Global Health Measures	Adverse events EQ-50 NASS Satisfaction Index NPRS PROMIS (PROMIS 10 or CAT) SANE VR-12	Yes
		Upper Extremity	ASES Standardized Shoulder Assessment DASH quickDASH OSS WOSI	
		Hip	HOOS, HOOS, Jr.	
		Knee	KOOS, KOOS Jr. MARS PEDI-IKDC	
		Foot and Ankle	FAAM FADI	
		Spine	ODI NDI	
Oberd – AO Global Data Registry	Allografts Scaffolds	Global Health Measures	PROMIS Short Form v1.0: Pain Interference 6b PROMIS Short Form v2.0: Physical Function 10a PROMIS Global Health SANE Visual Analog Scale	Yes
		Upper Extremity	ASES Shoulder Assessment QuickDASH PROMIS Short Form V2.0: Upper Extremity 7a	
		Hip	HOOS Jr.	
		Knee	KOOS Jr.	
		Foot and Ankle	FAAM	
		Spine	NDI ODI	
Code Technology	Allografts BMAC MFAT PRP Scaffolds	Global Health Measures	EQ-5D PROMIS Global 10 SF-36 VR-12	Yes
		Upper Extremity	ASES Shoulder Score DASH OSS PENN Shoulder Score SPADI UCLA Shoulder Score WOSI	
		Hip	AAOS Hip & Knee Score Harris Hip Score Hip Outcome Score HOOS, HOOS Jr. Oxford Hip Score	
		Knee	IKDC KOOS, KOOS Jr. Knee Society Score Lysholm Knee Scoring System MARS Oxford Knee Score WOMAC	
		Foot and Ankle	AOFAS FAAM	
		Spine	NDI ODI	
DataBiologics	A2M BMAC MFAT Plasma Lysate PPP PRP Prolotherapy Shock Wave Therapy	Global Health Measures	Adverse Events NPRS PHQ-4	Yes
		Upper Extremity	QuickDASH	
		Hip	HOOS Jr.	
		Knee	KOOS Jr.	
		Foot and Ankle	FAAM VISA-A	
		Spine	NDI ODI	
InCytes	Allografts BMAC Exosomes MFAT PRP Scaffolds Wharton’s Jelly	Global Health Measures	EQ-5D NPRS SF-12 SF-36 PROMIS VAS	Yes
		Upper Extremity	DASH QuickDASH PRTEE WORC, Short WORC WOSI	
		Hip	HOOS, HOOS Jr. iHOT-12 WOMAC	
		Knee	IKDC KOOS, KOOS Jr. Lysholm Knee Scoring Scale MOCART TAS VISA-P WOMAC	
		Foot and Ankle	FAAM FADI FFI MFPDI VISA-A	
		Spine	NDI ODI, ODI - Modified	
OutcomeMD	Allografts BMAC MFAT PRP Scaffolds	Global Health Measures	PROMIS	Yes
		Upper Extremity	ASES Shoulder Score IOF Wrist Fracture QuickDASH	
		Hip	HOOS Jr. mHHS	
		Knee	KOOS Jr.	
		Foot and Ankle	ATRS FAAm FFI-R	
		Spine	NDI ODI	
Amplitude Clinical Outcomes – International Cartilage Regeneration and Joint Preservation Society (ICRS)	Allografts Autologous anti-inflammatory injections BMAC MFAT PRP Scaffolds Stem Cell Amniotic-Based Injections	Global Health Measures	EQ-5D	Yes
		Knee	KOOS Kujala Anterior Knee Pain Scale	
Arthrex - Surgical Outcomes System	Allografts BMAC MFAT PRP Scaffolds	Global Health Measures	PROMIS-10 SANE VAS VR-12	Yes
		Upper Extremity	ASES-Elbow Score ASES Shoulder Score bMHQ CTS-6 KJOC Shoulder and Elbow Score Oxford Shoulder Score Penn Shoulder Score QuickDASH SST WORC WOOS WOSI	
		Hip	iHOT-12 mHHS NAHS Oxford Hip Score VHS	
		Knee	IKDC Knee Society Score Lysholm Knee Scoring System Knee Society Score KOOS, KOOS Jr. MARS MOCART Oxford Knee Score TAS	
		Foot and Ankle	AOFAS FAAM FFI-R MOCART	
		Spine	NDI ODI	
Ortech	Allografts BMAC MFAT PRP Scaffolds	Global Health Measures	EQ-50 PROMIS 10 or CAT SANE VR-12	Yes
		Upper Extremity	ASES Shoulder Score DASH QuickDASH Oxford Shoulder Score WOSI	
		Hip	HOOS, HOOS Jr.	
		Knee	Pedi-IKDC KOOS, KOOS Jr. MARS	
		Foot and Ankle	FAAM FADI	
		Spine	NDI ODI	
PatientIQ	BMAC PRP	Global Health Measures	EQ-50 PROMIS (PROMIS 10 or CAT) SANE VR-12	
		Upper Extremity	ASES Standardized Shoulder Assessment DASH quickDASH OSS WOSI	
		Hip	HOOS, HOOS, Jr.	
		Knee	KOOS, KOOS Jr. MARS PEDI-IKDC	
		Foot and Ankle	FAAM FADI	
DADOS	N/A	N/A	N/A	Yes
EUROSPINE – Spine Tango	Bone grafts Bone Morphogenetic Proteins Scaffolds	Global Health Measures	EQ-3D SF-36	Yes – European Equivalent
		Spine	COMI-Back NDI ODI SRS-30

**Table 2:** Summary of Registry Features and Costs

Registry/Data Collection Platform	Reminder Types	Compatible Devices	Costs	Partnerships & Sponsorships
Oberd – Regenerative Orthobiologics Registry (ROR)	Email Text Phone	Mobile Devices Tablets Personal Computers	$300 per provider per month	ROR
Oberd – AO Global Data Registry	Email Text	Mobile Devices Tablets Personal Computers	N/A	AAOS, AJRR, ASES, AO Foundation
Code Technology	Email Text Phone	Mobile Devices Tablets Personal Computers	Per provider and per procedure pricing models available	Over 27 organizations, including AAOS, AJRR, NASS, CJRR, MARCQI, Moon ACL Registry
DataBiologics	Email Text	Mobile Devices Tablets Personal Computers	$1500 per year	TOBI
InCytes	Email Text	Mobile Devices Tablets Personal Computers	Annual model: $3000 per year for up to 5 users and 100 cases overall; $5–10 per additional case Monthly model: $35 per month per account	
OutcomeMD	Email Text Phone	Mobile Devices Tablets Personal Computers	$95 per provider per month	ICHOM
Amplitude Clinical Outcomes – International Cartilage Regeneration and Joint Preservation Society (ICRS)	Email Text	Mobile Devices Tablets Personal Computers	$850 per provider per year	British Society for Surgery of the Hand, British Spine Registry, National Ligament Registry, ICRS
Arthrex - Surgical Outcomes System	Email	Mobile Devices Tablets Personal Computers	$200 per provider per month Free with membership to American Orthopedic Society for Sports Medicine, Irish Shoulder and Elbow Society, American Shoulder and Elbow Surgeons, Arthroscopy Association of North America, and Eastern Orthopedic Association	AANA, ASES, AOSSM, EOA
Ortech	Email Text	Mobile Devices Tablets Personal Computers	$6000 per provider for the first year $3000 per provider per year after	CJRR, AJRR, MARCQI, NASS, Alberta Arthroplasty Database, Nova Scotia Health Authority Joint Registry
PatientIQ	Email Text	Mobile Devices Tablets Personal Computers	N/A	AAOS Registry Program
DADOS	Email	Mobile Devices Tablets Personal Computers	N/A	
EUROSPINE – Spine Tango	Email Text	Mobile Devices Tablets Personal Computers	Free to any EUROSPINE member	EUROSPINE

Except for the InCytes data collection platform, all the other platforms highlighted in this review are partnered or sponsored by national or international registries. For example, the Oberd software has been used by the American Academy of Orthopaedic Surgeons (AAOS), American Joint Replacement Registry (AJRR), and American Shoulder and Elbow Surgeons (ASES). A summary of these partnerships can be found in Table 2. Overall, most of these registries have primarily collected data on orthopedic surgeries rather than orthobiologic treatments. However, there are some platforms that have a specific focus on the collection of PROMs for orthobiologic procedures. Oberd was utilized to create the Regenerative Orthobiologics Registry (ROR) [23, 24]. DataBiologics was recently endorsed as the official outcomes software for The Orthobiologic Institute (TOBI) [25, 26]. InCytes was created with a focus on collecting data for orthobiologic procedures [27]. Amplitude Clinical Outcomes is the software that powers the International Cartilage Regeneration and Joint Preservation Society (ICRS), which is another registry that focuses on orthobiologics [28, 29].

All the data collection platforms can be used to record the diagnosis for the treatment, the type of orthobiologic procedure performed, company and product name of the orthobiologic system, and where the treatment was performed. Besides the Spine Tango registry which only collects data for treatments of spine diseases and the ICRS which only collects data for knee pathologies, all the platforms collect PROMs for treatments across different parts of the body. A summary of the PROMs used for specific joints for each registry can be found in Table 1 [24, 25, 27–38]. Most of the data collection platforms have pre-set follow-up periods for sending PROMs, such as 3-months, 6-months, 9-months, one year, and two years. The Code Technology, InCytes, OutcomeMD, and Ortech platforms also allow clinicians to create customizable follow-up periods according to the organization’s needs.

Another important consideration in the type of PROMs utilized is response burden. A Cochrane review by Edwards et al. concluded that questionnaire length has a substantial impact on non-response rates. Amongst 56 trials, the odds of response increased by more than half using shorter postal questionnaires (OR 1.64, 95% 1.43–1.87). Only two trials involving electronic questionnaires were included in this same Cochrane review, but the odds increased by over a half when using shorter questionnaires (OR 1.73, 95% CI 1.40 to 2.13). However, this review noted that although shorter questionnaires were found to minimize non-response, it may be at the cost of a reduction of accuracy of the measurement process [39]. Thus, shortening questionnaires may not be an option unless if there have been studies to validate the abridged PROM. Examples of validated shortened versions of PROMs that these data collection platforms utilize include the QuickDASH, HOOS Jr., and KOOS Jr. [24, 25, 27–38]. Furthermore, the Patient Reported Outcome Measurement Information System (PROMIS) is a tool that can reduce response burden while maintaining validity of its measures. PROMIS utilizes computer-adaptive tests (CAT) where computer assessment software can deliver a brief and targeted sequence of items to an individual based on his or her previous item response. A typical PROMIS CAT can involve four to eight items and take about one to two minutes to complete while maintaining validity [40]. Data collection platforms that utilize PROMIS CAT include ROR, AO Global Data Registry, Code Technology, InCytes, OutcomeMD, Surgical Outcomes System, Ortech, and PatientIQ [27, 30–34, 38].

There are additional measures that can be taken to improve response rates. Pre-operative participation in PROM surveys had a significant positive association with participation at 3 months (OR 3.34, 95% CI 2.76–4.04) and at 1-year (OR 15.46, 95% CI 12.16–19.67) [41]. Compared to paper forms, electronic and Web-based methods of PROM collection has also shown to be more effective and has led to higher completion rates [42, 43]. Other solutions that also improved post-treatment survey completion included an electronic dashboard to track patients who did not complete their questionnaires and re-sending surveys, providing a paper version, and calling the patient to follow-up [44]. All the data collection platforms included in this review emphasize collecting pre-treatment PROMs. Email and text reminders are also utilized by all the data collection platforms, except for Arthrex which does not have text messaging reminders. ROR, Code Technology, and OutcomeMD also offer phone call reminders to patients. Furthermore, these registries are all web-based platforms where patients can complete PROMs on different devices, such as smartphones, tablets, and personal computers. Through these registries, clinicians will also have access to dashboards to track completion rates of PROMs. Some platforms, such as Code Technology, also have account managers who help to keep track of PROM completion rates [24, 25, 27–38].

Data privacy can also be a concern to both providers and patients. All the registries described in this review are HIPAA compliant or follow similar regulations based on their country of origin. These registries also utilize strict security protocols to ensure that patient data is protected. Costs of the data collection platform is another important factor that clinicians may consider. A summary of costs can be found in Table 2. However, pricing may vary depending on different factors, including choice of pricing models, licensing fees, specific needs and size of the institution, and additional features. Pricing information for certain data collection platforms were also not publicly disclosed and required an onboarding demonstration. Additionally, InCytes charged a fee per additional case after a maximum of 100 cases were reached. Code Technology also offered either a per provider or per procedure payment model. Fees per procedure or case could cause a potential selection bias when recruiting patients into the registry. Both the Surgical Outcomes System and Spine Tango registries can be utilized for free with membership to certain medical societies or groups [24, 25, 27–38].

## Discussion

The literature on the use of orthobiologics has been mixed. Some recent RCTs and case series have helped to support the use of orthobiologic treatments [14–18]. For example, PRP was shown to be superior to hyaluronic acid or saline solution in the treatment of mild to moderate knee osteoarthritis [16]. However, other studies found that PRP did not provide a superior clinical improvement compared with hyaluronic acid [45, 46]. Study design could contribute to these mixed results, such as lack of standardization and characterization of the orthobiologic used in the study. Additionally, many of these trials contain small sample sizes, short duration of follow-up, and large costs and resources to perform [14–18]. Due to these limitations of RCTs, literature regarding adverse events and long-term follow up of orthobiologic treatments is lacking [47]. Registry data can be collected quickly and efficiently to help supplement the existing literature. Additionally, long-term outcomes of large patient populations can be collected through patient registries to capture rare and serious adverse events of orthobiologics that may not be detected during RCTs that are limited in time and sample size [48]. Currently, there is also a lack of literature on how the characteristics of the orthobiologic procedure can affect treatment, such as the volume or constituents of the injected material [47]. Patient registries could also be used to help fill this gap of knowledge.

The data collection platforms in this review gather important information, such as diagnosis, type of orthobiologic procedure performed, company and product name of the orthobiologic system, and where the treatment was performed [24, 25, 27–38]. However, clinicians should provide feedback to these data collection platforms to allow more specific parameters of the treatment to be entered into the platforms, such as volume of injectate and concentration of cellular components. All the patient registries in this review provide data analysis and outcome reports based on diagnoses and treatments. Having outcomes data on the specific parameters of treatments could help clinicians determine their efficacy and support decision making on how to best optimize orthobiologic procedures. Furthermore, long-term outcomes data collected through patient registries could help facilitate communication regarding the treatment course and prognosis of orthobiologics [49]. Finally, patient registry data can also be used to help determine the cost-effectiveness of orthobiologics [1].

Overall, the data collection platforms discussed in this review can help clinicians collect and monitor outcome measures. These platforms provide a system and interface that allows clinicians to easily collect data from patients without having to develop and design their own software, thus saving clinicians resources on time and money. Additionally, these platforms have an onboarding process that provides training in optimizing the use of the software as well training staff on best use practices in implementing a patient registry. By automating patient reminders to complete patient questionnaires, clinicians and their staff would not need to dedicate as much effort on patient follow-up. Furthermore, these platforms can be used as an effective feedback tool by providing data analysis and outcome reports to allow clinicians to make choices on how to improve and change treatment protocols based on their specific patient populations.

### Review limitations

One disadvantage of registry data is lack of a control group. Thus, it is important to utilize patient registries to supplement data and optimize orthobiologic treatments that have been supported through RCTs. Additionally, there is concern of non-response bias with patient registries [50]. However, as previously discussed, all the data collection platforms in this review utilize various measures to increase response rates, including being internet-based platforms, collecting pre-treatment PROMs, automated patient reminders, and utilizing shorter questionnaires to reduce response burden [24, 25, 27–38]. Although all the patient registries in this review provide data analyses and reports, such as graphs with trends of the data, the patient registries in this review are not subjected to obligatory third-party checks or independent audits [51]. Thus, the data could be subject to random and systematic errors [52].

A limitation of this review is that it might not be possible to have identified all orthopedic registries. Although a robust search was attempted, registries that collect PROMs for orthobiologic procedures may have been missed as new platforms are continuing to be developed and started. Additionally, information regarding registry systems could be limited to only what is available publicly online. Attempts were made to directly contact the vendors discussed in this review. However, some companies were unable to be contacted. Other companies required a demonstration or onboarding process prior to disclosing specific information, such as pricing. Information regarding costs to utilize these registry systems is also limited as pricing depends on each institution’s needs, such as size of the institution, additional features offered, licensing fees, and other variables.

### Future perspective

There are currently various orthopedic surgery registries available to clinicians. These registries also collect data on orthobiologic treatments but is not the main objective of these patient registries. However, patient registries specific to the collection of outcomes related to orthobiologic procedures are quite limited and include ROR, ICRS, DataBiologics, and InCytes. Furthermore, orthobiologic-specific patient registries have published sparse data mostly in the form of annual reports. According to The ICRS Patient Registry Annual Report 2020, no complications were documented following injection with orthobiologic treatments [29]. In the 2021 Outcomes Report for DataBiologics, meaningful reduction in pain within 12-months was found in 73, 70, and 61% of patients with knee osteoarthritis following treatment with PRP, adipose tissue therapy, and BMAC respectively. This report concluded that their outcomes data demonstrated potential effectiveness of PRP and adipose tissue based treatments for knee osteoarthritis [53]. Ultimately, the continued use and support of these patient registries is needed to allow for the growth of more outcomes data of orthobiologic procedures. With more outcomes data, the cost-effectiveness of orthobiologics can be determined. By using registry data to perform cost-benefit analyses, orthobiologic procedures could hopefully be proven to be the standard of care for specific indications and accepted by medical insurers.

## Conclusions

Orthobiologic procedures are becoming more frequently performed clinically although there are concerns regarding their safety, clinical efficacy, and limited available evidence. Collection of PROMs can provide data on pain and function outcomes as well as elucidate the long-term safety, effectiveness, and potential cost savings of orthobiologic procedures. Various data collection platforms and registries have been described and compared in this review. Each platform offers different services to facilitate in data collection and vary in pricing. Clinicians have different goals when considering which registry to adopt into their practice. Furthermore, many clinicians have busy practices with limited resources or capabilities to independently employ a database registry in their practices. Thus, there appears to be a need for affordable and easy to use database registries specifically for the collection of outcomes for orthobiologic procedures. Providing this comparison will hopefully aid physicians in choosing a platform to collect PROMs.

## Data Availability

Data sharing is not applicable to this article as no datasets were generated or analyzed during the current study. Not applicable.

## Abbreviations

A2M: Alpha-2 Macroglobulin
AANA: Arthroscopy Association of North America
AAOS: American Academy of Orthopaedic Surgeons
AJRR: American Joint Replacement Registry
AOFAS: American Orthopedic Foot and Ankle Score
AOSSM: American Orthopedic Society for Sports Medicine
ASES: American Shoulder and Elbow Surgeons
ATRS: Achilles Tendon Total Rupture Score
BMAC: Bone Marrow Aspirate Concentrate
bMHQ: Brief Michigan Hand Outcome Questionnaire
CAT: Computer Adaptive Test
CJRR: California Joint Replacement Registry
COMI-Back: Core Outcome Measures Index for the Back
CTS-6: 6-Item Carpal Tunnel Symptoms Scale
DASH: Disabilities of the Arm, Shoulder, and Hand
EOA: Eastern Orthopedic Association
EQ: European Quality of Life
EurQol: European Quality of Life
FAAM: Foot and Ankle Ability Measure
FADI: Foot and Ankle Disability Index
FFI-R: Foot Function Index Revised
FORCE-TJR: Function and Outcomes Research for Comparative Effectiveness in Total Joint Replacement
GHLF: Global Healthy Living Foundation
HOOS: Hip Disability and Osteoarthritis Outcome Score
ICHOM: International Consortium of Health Outcome Measures
ICRS: International Cartilage Regeneration and Joint Preservation Society
iHOT-12: International Hip Outcome Tool 12-Item
IKDC: International Knee Documentation Committee
IOF: International Osteoporosis Foundation
ISAR: International Society of Arthroplasty Registries
KJOC: Kerlan-Jobe Orthopaedic Clinic
KOOS: Knee Injury and Osteoarthritis Outcome Score
MARCQI: Michigan Arthroplasty Registry Collaborative Quality Initiative
MARS: Marx Activity Rating Scale
MFAT: Microfragmented Adipose Tissue
MFPDI: Manchester Foot and Pain Disability Index
mHHS: Modified Harris Hip Score
MOCART: Magnetic Resonance Observation of Cartilage Repair Tissue
NAHS: Non-Arthritic Hip Score
NASS: North American Spine Society
NDI: Neck Disability Index
NIH: National Institutes of Health
NPRS: Numeric Pain Rating Scale
NSQIPs: National Surgical Quality Improvement Programs
NTDB: National Trauma Data Bank
ODI: Oswestry Disability Index
OSS: Oxford Shoulder Score
Patient Reported Outcome Measures: PROMs
Pedi-IKDC: Pediatric International Knee Documentation Committee Subjective Knee Form
PHQ-4: Patient Health Questionnaire-4
PPP: platelet-poor plasma
PROMIS: Patient-Reported Outcomes Measurement Information System
PRP: platelet-rich plasma
PRTEE: Patient-Related Tennis Elbow Evaluation
RCT: Randomized Control Trial
ROR: Regenerative Orthobiologics Registry
RWS: Randall Woolcott Services
SANE: Single Assessment Numeric Evaluation
SF-36: 36-Item Short Form Survey
SOS: Surgical Outcomes System
SPADI: Shoulder Pain and Disability Index
SRS-30: Scoliosis Research Society 30-Item
SST: Simple Shoulder Test
TAS: Tegner Activity Scale
TJRR: Total Joint Replacement Registry
UAB: University of Alabama at Birmingham
VAS: Visual Analog Score
VHS: Vail Hip Score
VISA-A: Victorian Institute of Sports Assessment – Achilles
VISA-P: Victorian Institute of Sports Assessment – Patellar Tendinopathy
VR-12: Veterans RAND 12
WOMAC: Western Ontario and McMaster Universities Osteoarthritis Index
WOOS: Western Ontario Osteoarthritis Shoulder Index
WORC: Western Ontario Rotator Cuff Index
WOSI: Western Ontario Shoulder Instability Index
